# Development of a Pilot Literacy Scale to Assess Knowledge, Attitudes, and Behaviors towards Climate Change and Infectious Disease Dynamics in Suriname

**DOI:** 10.3390/ijerph20247178

**Published:** 2023-12-14

**Authors:** Meghan Matlack, Hannah Covert, Arti Shankar, Wilco Zijlmans, Firoz Abdoel Wahid, Ashna Hindori-Mohangoo, Maureen Lichtveld

**Affiliations:** 1 School of Medicine, University of North Carolina at Chapel Hill, 321 S Columbia Street, Chapel Hill, NC 27514, USA; 2 Department of Environmental and Occupational Health, University of Pittsburgh School of Public Health, 130 De Soto Street, Pittsburgh, PA 15261, USA; hcovert@pitt.edu (H.C.); fza3@pitt.edu (F.A.W.); mlichtve@pitt.edu (M.L.); 3 Department of Biostatistics and Data Science, Tulane University School of Public Health and Tropical Medicine, 1440 Canal Street, New Orleans, LA 70112, USA; sarti@tulane.edu; 4 Faculty of Medical Sciences, Discipline of Pediatrics, Anton de Kom University of Suriname, Leysweg 86, Paramaribo P.O. Box 9212, Suriname; wilco.zijlmans@uvs.edu; 5 Foundation for Perinatal Interventions and Research in Suriname (Perisur), Anton Dragtenweg 93, Paramaribo, Suriname; ashna.mohangoo@perisur.org

**Keywords:** climate change, infectious disease, vector-borne disease, literacy, knowledge, education

## Abstract

Prior research has shown that climate literacy is sparse among low- and middle-income countries. Additionally, no standardized questionnaire exists for researchers to measure climate literacy among general populations, particularly with regards to climate change effects on vector-borne diseases (VBDs). We developed a comprehensive literacy scale to assess current knowledge, attitudes, and behaviors towards climate change and VBD dynamics among women enrolled in the Caribbean Consortium for Research in Environmental and Occupational Health (CCREOH) cohort in Suriname. Items were generated by our research team and reviewed by a group of six external climate and health experts. After the expert review, a total of 31 climate change and 21 infectious disease items were retained. We estimated our sample size at a 10:1 ratio of participants to items for each scale. In total, 301 women were surveyed. We validated our scales through exploratory (*n* = 180) and confirmatory factor analyses (*n* = 121). An exploratory factor analysis for our general Climate Change Scale provided a four-construct solution of 11 items. Our chi-squared value (X^2^ = 74.32; *p* = 0.136) indicated that four factors were sufficient. A confirmatory factor analysis reinforced our findings, providing a good model fit (X^2^ = 39.03; *p* = 0.23; RMSEA = 0.015). Our Infectious Disease Scale gave a four-construct solution of nine items (X^2^ = 153.86; *p* = 0.094). A confirmatory factor analysis confirmed these results, with a chi-squared value of 19.16 (*p* = 0.575) and an RMSEA of 0.00. This research is vitally important for furthering climate and health education, especially with increases in VBDs spread by Aedes mosquitoes in the Caribbean, South America, and parts of the southern United States.

## 1. Introduction

Climate change is a concept that is well-known and well-researched across the world, especially in high-income countries such as the United States [1,2]. However, climate change literacy rates vary substantially across the world. In low- and middle-income countries (LMICs), climate literacy rates tend to be much lower compared with high-income nations [3,4]. In a 2015 study published in Nature Climate Change, which assessed the results of a 2007–2008 World Gallup poll, participants across 119 countries were asked about their knowledge of global warming or climate change [3,4]. This study found that participants from high-income countries were more likely to say they were aware of climate change, with awareness rates of over 90% across North America and Europe [3]. Conversely, many LMICs had climate change awareness rates of 50% or below [3]. In Africa, LMICs like Tunisia and Mozambique have climate literacy rates as low as 23% and 25%, respectively [5].

Although inadequate in several parts of the world, climate change literacy is important for several reasons. Widespread climate education is essential to help the general population better understand and relate to climate change issues and can allow them to make better-informed personal and community health decisions to reduce their climate impact and undertake adaptive behaviors [6,7]. Improved knowledge of climate change and the associated consequences is also an important driver for the successful implementation of individual pro-climatic behaviors, especially among younger populations [8].

At present, there are little to no assessments on climate change literacy for populations living in LMICs and even less so in the Caribbean. In our prior review [9], we found a limited number of knowledge, attitude, and practice (KAP) studies in LMICs; only one study focused on climate change literacy in the Caribbean, specifically in Jamaica [10]. Small-island developing states like those in the Caribbean are increasingly susceptible to changes in temperature and precipitation patterns, paving the way for increases in extreme weather events such as heatwaves, cyclones, tropical storms, droughts, sea level rises, and flooding [6,11,12]. Climate change will not only make these events more frequent, but will also intensify them, causing widespread environmental and human health impacts such as increases in infectious diseases, heat-related morbidity and mortality, flood- and storm-induced infrastructure damage, and water and food scarcity [11]. Infectious diseases are a particular concern with regards to climate change. Because of increased temperatures and changes in precipitation patterns, vector-borne disease incidence has risen as vector ranges have expanded or vector reproduction has increased [11]. These concerns are even more evident in the Caribbean region, which has experienced rapid increases in mosquito-borne disease outbreaks (namely, dengue, Zika, and chikungunya) over the past 30 years [13]. At present, infectious disease literacy is also limited and does not effectively evaluate VBD and climate change interactions, particularly in more affected regions such as the Caribbean [9].

Our current study aims to rectify the limited availability of current climate change and infectious disease literacy assessments in the Caribbean region through the development of a comprehensive literacy scale evaluating knowledge, attitudes, and behaviors of women currently enrolled in the Caribbean Consortium for Research in Environmental and Occupational Health (CCREOH) cohort [14] with regards to climate change and infectious disease dynamics in Suriname. Utilizing the expertise obtained from our prior scoping review [9], we created a scale that covered topics such as food and water availability, droughts and heatwaves, hurricanes and extreme weather events, sea level rises and flooding, mosquito ecology and transmission, preventive actions against mosquito-borne diseases, and government and media influence. Our central goal in the creation of this literacy scale was to provide an effective and generalizable instrument to evaluate current climate change and infectious disease knowledge, attitudes, and behaviors that could be applied across a variety of populations in the Caribbean region. We hope to utilize our findings from this work to strengthen future vector-borne disease interventions at both the individual and community levels.

## 2. Materials and Methods

### 2.1. Study Area

Suriname is located on the coast of northeastern South America and is bordered by Guyana, French Guiana, Brazil, and the Atlantic Ocean. Eligible participants were sampled from either the highly urban capital of Paramaribo or the more rural and agriculturally rich district of Nickerie [15], both located along the coastal zone. Approximately 80% of Suriname’s population lives within this coastal zone, with the rest of the population, primarily Indigenous and Tribal Peoples, occupying the rural interior [16].

### 2.2. Sample Size and Participant Selection

In accordance with common guidelines that recommend between 5 and 10 participants per item [17,18], we estimated our sample size based on an approximate 10:1 ratio, with 30 total scale items per scale and 10 participants per item, making our initial sample size 300 participants. We then calculated a 30% oversampling rate to account for any participant withdrawals or non-responses, bringing our total potential sample size to 390. Our eligible participant sample consisted of approximately 624 adult women from the CCREOH cohort that lived in either Paramaribo, Nickerie, or the surrounding districts. We implemented a 70–30% split of the sample (as more of the cohort came from Paramaribo and Paramaribo-adjacent districts compared with Nickerie) and generated a simple random sample to determine which participants would be contacted for participation. This gave us a total of 273 eligible participants selected from Paramaribo and 117 eligible participants selected from Nickerie for data collection.

### 2.3. Scale Development

We utilized readily available questionnaires [19,20,21,22,23,24,25] from our previous scoping review [9] to provide guidance for the creation of our own literacy items. Our research team then generated a more extensive list of 119 original potential literacy scale items that included several important climate change and infectious disease topics such as temperature and rainfall effects on climate change, sea level rises and the warming of oceans, mosquito ecology and disease transmission, potential infectious disease risks and prevention methods, and media and government influence. Each literacy item developed by our team was categorized into one of three domains: knowledge, attitude, or behavior. By paraphrasing definitions from a prior environmental literacy scale created by our research team, we defined knowledge as climate change and infectious disease facts or information that was previously gained through education or personal experience [26]. Attitudes were defined as ways of thinking about or perceiving climate change or infectious disease literacy [26]. Behaviors were defined as ways that individuals act in response to climate change or infectious disease concerns [26]. We created two separate scales, one to assess general climate change topics, and another to specifically assess mosquito-borne disease dynamics as a consequence of climate change. Of these items, 64 belonged to the general climate literacy scale and 55 belonged to the mosquito-borne disease scale. Items were written as declarative statements and all scale items were written to reflect a 5th grade reading level. Knowledge and attitude items followed a five-point Likert scale response of strongly agree (5), agree (4), don’t know (3), disagree (2), and strongly disagree (1). Behavior items followed a five-point frequency scale response of always (5), frequently (4), sometimes (3), rarely (2), and never (1). Once the scale item development was completed, the drafted items were sent to an outside panel of six climate and health experts; four were experts specifically in the Caribbean region, who then reviewed each potential scale item and rated them as either ‘essential to include’, ‘useful but not essential to include’, or ‘not needed’. Reviewers also rated items based on which domain they best fitted under. We first retained only items that had at least 80% or greater reviewer agreement for an ‘essential’ rating, leaving us with 40 items; 26 were retained for the general Climate Change Scale and 14 were retained for the mosquito-borne diseases scale. This provided us with at least three items for each domain across both scales (Supplementary Materials, Tables S1 and S2). To capture additional items for the factor analysis, we also retained scale items that were classified by our expert reviewers as either ‘essential’ or ‘useful but not essential’ and had a percent agreement score of at least 67% (4 out of 6 reviewers). With this method, we retained an additional 12 items that were suitable to include in our analysis. In total, there were 52 items after the expert review that were included in our final literacy scale. The items chosen for retention for both scales prior to the exploratory and confirmatory analyses can be found in Supplementary Materials, Table S3. A complete version of the scales, including a demographic questionnaire, can also be found in the Supplementary Materials (File S1).

### 2.4. Data Collection

Eligible participants were notified of our study over the phone and asked to attend in person to take our questionnaire. We obtained approval for our research protocol from both the University of Pittsburgh’s Institutional Review Board (Study 22090104) as well as the Medical Ethical Committee of the Ministry of Health in Suriname (reference number VG 023–14). The questionnaire was completely voluntary and verbal consent was obtained from all participants prior to the survey administration. Eligible participants were given paper-based questionnaires and surveyed by Dutch-speaking recruiters. Questionnaire completion was estimated at 30–45 min, and all responses were recorded using REDCap. The survey was administered to 301 participants over the course of approximately 8 weeks, from May 2023 to July 2023.

### 2.5. Statistical Analysis

**Figure A1:**
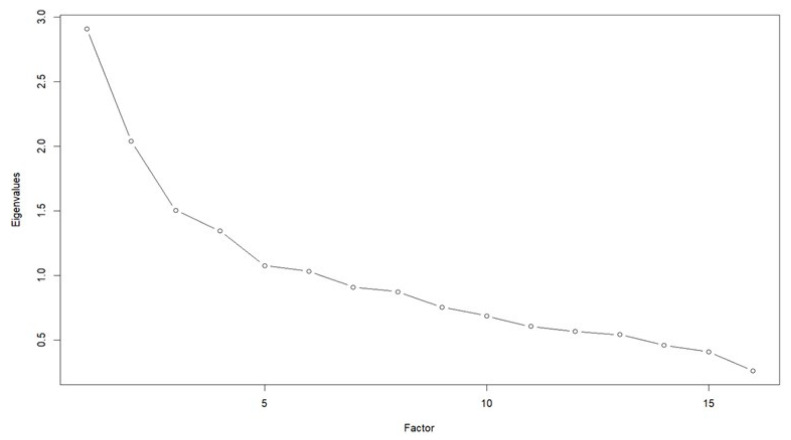
Scree plot of factors for the EFA for the Climate Change Scale, iteration 3. Based on where the largest drop occurred before leveling out (*n* = 5), we retained the four factors prior to this and dropped the others. Sufficiency of four factors was confirmed with a chi-squared test.

**Figure A2:**
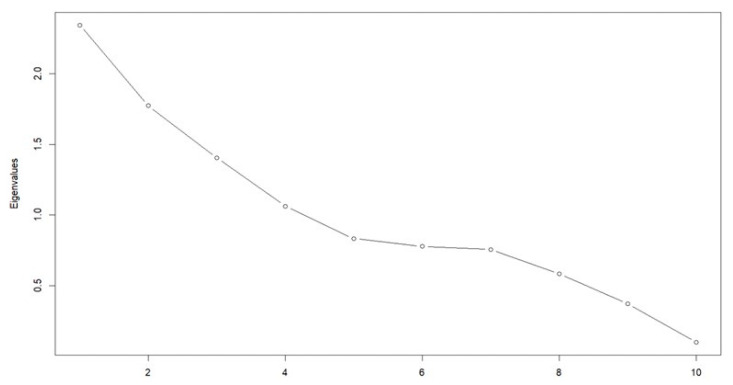
Scree plot of factors for the EFA for the Infectious Disease Scale, iteration 2. Based on where the largest drop occurred before leveling out (*n* = 5), we retained the four factors prior to this and dropped the others. Sufficiency of four factors was confirmed with a chi-squared test.

We used exploratory (EFA) and confirmatory factor analyses (CFA) to evaluate the psychometric properties of both the climate change and vector-borne disease (VBD) scales. Items were first separated based on which scale they belonged to, then the data were randomly split using a 60:40 split, with 180 observations used for EFA and 121 used for CFA. Participant demographics for both the exploratory and confirmatory samples can be found in Table 1. Factors were retained based on scree plots (Appendix A, Figure A1 and Figure A2), the amount of variance explained, and chi-squared tests to determine the sufficiency of factors. Items were assessed based on their factor loadings as follows. Items with factor loadings greater than 0.40 were classified as fair, items with factor loadings greater than 0.55 were classified as good, and items with loadings greater than 0.71 were classified as excellent [27]. Based on these values and a general rule-of-thumb [28,29,30], any item with less than a 0.4 loading on any factor was excluded from further analyses. Items with high cross-loadings (>0.25) were interpreted as being associated with one or more factors and these items were dropped through our EFA iterations to ensure that the retained items loaded highly onto one factor only. However, this was not an issue in our analyses as the rotation of our factors eliminated most, if not all, of our cross-loadings. The cross-loadings that remained fell under the 0.25 threshold we set based on previous work [26]. Prior literature suggests at least three items per factor are required [31,32,33]; the initial EFA iterations were unable to meet this guideline, so we chose to retain factors with at least two items for further analyses. Factors that had fewer than two items were not included in any further analyses [34,35].

Maximum likelihood was used to estimate the parameters for the CFA. Our CFA models were assessed using a variety of tests, including a chi-squared test and root mean square error of approximation (RMSEA) to determine the model fit. Our RMSEA cutoff value was 0.1. Our team’s prior environmental health literacy scale also utilized an RMSEA of 0.1 [26]. The internal consistency and reliability of the items for both scales were assessed using Cronbach’s alpha. The cutoff reliability was established to be 0.70. Our hypotheses were all tested at a significance level of 0.05 and all analyses were completed using R Statistical software v4.2.2, R Core Team 2022 [36].

## 3. Results

### 3.1. Climate Change Literacy Scale

We conducted an EFA on the initial 31-item Climate Change Scale to examine the underlying factor structure. Three iterations of the EFA were necessary to reach a factor solution that was interpretable. The final EFA resulted in a four-construct solution with a total of 11 items (Table 2).

Factors were selected based on the specific constructs they described. Item loadings ranged from −0.183 to 0.903, and no cross-loadings exceeded our 0.25 threshold. The chi-squared test evaluating the underlying factor structure was not significant (X^2^ = 74.32; *p* = 0.136), demonstrating that our four-factor solution was sufficient. Factor 1 (general climate effects) explained 11.6% of the total variance, Factor 2 (heat) explained 9.7% of the total variance, Factor 3 (indoor cooling) explained 8.6% of the total variance, and Factor 4 (ocean warming) explained 7.4% of the total variance. The cumulative variance explained by the four constructs was 37.2%. Eigenvalues for the retained factors are shown in Table 3. Inter-factor correlations for all four constructs are shown in Appendix A, Table A1. We conducted a CFA on our Climate Change Scale using the confirmatory test sample of participants (*n* = 121). Our chi-squared test (X^2^ = 39.03; *p* = 0.23), along with our root mean square error of approximation (RMSEA) value (0.015), indicated a very good fit of the items. Overall, Cronbach’s alpha was 0.47, which was below our cutoff of 0.7. The Cronbach’s alphas calculated per factor were as follows: for Factor 1 (general climate effects), Cronbach’s alpha was 0.68; for Factor 2 (heat), Cronbach’s alpha was 0.52; for Factor 3 (indoor cooling), Cronbach’s alpha was 0.54; and for Factor 4 (ocean warming), Cronbach’s alpha was 0.82. Additional measures of goodness of fit can be found in Appendix A, Table A3.

### 3.2. Infectious Disease Literacy Scale

We conducted two iterations of the EFA to obtain an interpretable factor solution for our 21-item Infectious Disease Scale. The final EFA resulted in a four-factor solution with a total of nine items (Table 4).

Item loadings ranged from −0.186 to 0.996. No cross-loadings were over 0.25. Our four-factor solution was sufficient, as evidenced by the chi-squared test (X^2^ = 153.86; *p* = 0.0937). Factor 1 (disease transmission) explained 9.4% of the total variance, Factor 2 (temperature effects) explained 6.9% of the total variance, Factor 3 (viruses) explained 6.4% of the total variance, and Factor 4 (water-holding containers) explained 5.9% of the total variance. The cumulative variance explained by all four factors was 28.5%. Eigenvalues for the retained factors are shown in Table 5. Inter-factor correlations for the four constructs are shown in Appendix A, Table A2. Our CFA results for the Infectious Disease Scale also showed a very good model fit (X^2^ = 19.16; *p* = 0.575; RMSEA = 0.00). Cronbach’s alpha was 0.45, which was below our cutoff of 0.7. Individual Cronbach’s alphas for each factor were calculated as follows: Factor 1 (disease transmission) had a Cronbach’s alpha value of 0.38, Factor 2 (temperature effects) had a value of 0.80, Factor 3 (viruses) had a value of 0.67, and Factor 4 (water-holding containers) had a value of 0.95. Additional measures of goodness of fit can be found in Appendix A, Table A3.

## 4. Discussion

The goal of this study was to develop a validated scale of climate change and VBD constructs to adequately assess literacy about these issues among women enrolled in the CCREOH cohort based in Suriname. Our survey instrument utilized five-point Likert scales to evaluate current participant knowledge, attitudes, and behaviors towards climate change and climate–VBD interactions. Our final scale contained both a general climate change section as well as a VBD-specific section and had a total of 20 items that were created through our EFA and CFA results.

Our 11-item Climate Change Scale described a four-construct solution as follows: general climate change effects, containing three items; heat effects, containing four items; staying indoors/household cooling, containing two items; and ocean warming, containing two items. Together, these factors explained 37.2% of the total variability. Internal reliability was measured using Cronbach’s alpha, which was 0.47 and below our cutoff of 0.7. A calculation of Cronbach’s alpha for individual factors yielded slightly improved results, although our second and third factors still produced alpha values below our 0.7 cutoff. Our nine-item Infectious Disease Scale described a four-construct solution as follows: disease transmission, containing three items; temperature effects, containing two items; viruses, containing two items; and water-holding containers, containing two items. These four factors explained 28.5% of the total variability. Cronbach’s alpha for our Infectious Disease Scale was 0.45, which was also below our cutoff of 0.7. Individual Cronbach’s alpha values for these factors greatly improved, although the alpha value for Factor 1 (disease transmission) was much lower than the overall alpha value calculated. Both scales had otherwise good-fit statistics (Appendix A, Table A3) and good absolute model fits (RMSEA = 0.015 for the Climate Change Scale; RMSEA = 0.00 for the Infectious Disease Scale).

In assessing our raw survey data, we found that most participants agreed that climate change was primarily anthropogenic and unavoidable, while approximately 28% of those surveyed were unsure or did not agree that climate change would affect future generations. Preventive measures among this population were also inconsistent. Fewer than half of all participants reported frequently or always staying indoors during heatwaves, although 70.7% affirmed frequently or always keeping their homes as cool as possible during heatwaves. This specific result may have had a higher agreement due to the fact that Suriname has a hot, tropical climate and consistently experiences temperatures between 70 and 90 degrees Fahrenheit [37,38], and people are more likely to keep their homes cooled regardless of their level of climate change literacy. There was a high agreement for the two ocean-warming items included (88.1% and 87.7%, respectively, for items 10 and 11 in the Climate Change Scale (Table 2). Commercial fishing in Suriname is incredibly important for the livelihoods of many individuals as it can provide income through the export of fish and other marine animals such as shrimp [39]. Previous research has shown that increased ocean temperatures are likely to negatively impact fish populations by reducing reproductive output and thus limiting their ability to successfully repopulate [40,41], which poses real problems for both local and commercial fishers.

Our Infectious Disease Scale retained constructs surrounding disease transmission, higher temperatures, viruses, and prolonged water storage. Survey respondents were somewhat aware of the association between temperature and VBDs, with approximately 55% of participants agreeing that warmer temperatures would also increase VBD transmission. The association between precipitation and VBDs was much more pronounced, with over 80% of participants reporting that changes in precipitation would increase VBD transmission. In addition, 81.1% of participants agreed that VBD outbreaks occur more often during rainy seasons. Precipitation projections for Suriname specifically predict less-frequent rainfall but more severe episodes of precipitation over the next several decades [42]. It is imperative to note that these future episodes are much more likely to increase mosquito habitats in and around water-holding containers. Our respondents tended to already be very aware of these water-storage risks with regards to VBDs as over 75% of the participants surveyed frequently or always removed water from open storage containers and/or removed open storage containers themselves. However, participants were less likely to believe that they personally had increased VBD risks compared with the rest of their community, and most reported seeing no recent change in VBD outbreaks within their areas.

Additional climate change and infectious disease constructs that were evaluated using our survey instrument but not ultimately retained for our final scales included sea level rise, extreme weather, access to food, preventive measures and activities, mosquito ecology, and access to information and media sources. The items that described these constructs (20 of 31 for the Climate Change Scale; 12 of 21 for the Infectious Disease Scale) were all dropped during the EFA. We found that these items either did not load highly (<0.4) onto any factor during our EFA iterations or could not provide an interpretable factor solution. It could be that these concepts were not retained due to poorly worded items or items that may have been too specific in their wording, which was highlighted in the fact that our EFA was unable to identify an underlying KAP structure as expected and thus our scales were instead defined by these narrowly defined constructs, which limited our analyses.

Further exploration of these topics is required, although the scales presented in this study provide a strong pilot tool for researchers. First, the overall small sample size was a limiting factor in our analyses. We estimated that about 300 participants would be required, based on a prior guideline suggesting that 10 participants per item should be included in a scale for factor analysis [17,18]. Although we met this criterion overall, we had to split this sample for the exploratory and confirmatory analyses, leaving 180 observations for the EFA and 121 for the CFA. Further, several of our constructs among both scales retained only two items after the EFA, whereas a minimum of three items per factor is recommended for optimal analyses [31,32,33]. An overall larger sample size may have yielded different results and given us a higher number of items retained per scale. Additionally, our current sample only included women as our sample was obtained from CCREOH, an existing maternal and child cohort. Thus, our sample was limited to responses from one specific population and will need to be rectified in future iterations. Our Cronbach’s alpha values for both the Climate Change and Infectious Disease Scales were also lower than expected, with low internal consistency. This could indicate that our scales may have had several items that were less highly interrelated; however, this was considered to be a minor problem when conducting our EFAs and when trying to load items to specific factors. This was mitigated by calculating Cronbach’s alpha values per individual factors. Because of the timeframe, information on some important variables could not be collected and so we could not measure divergent and convergent validity. We were also unable to obtain a three-factor solution based on our initial domains (knowledge, attitudes, and behaviors). The preliminary EFA on the Climate Change Scale gave a solution with three factors but had no high item loadings for ‘behavior’ on any factor and could not be used. Many of the ‘behavior’ items had negative loadings on our factors. We hypothesized that since some of the ‘behavior’ concepts that we included were not widely known or practiced within the community surveyed, this may potentially explain why none of our ‘behavior’ items were able to highly load onto one factor. In particular, ‘behavior’ items pertaining to water-saving strategies, emergency preparedness, and mosquito intervention methods were the items most likely to have very low practice rates. A cultural adaptation of the literacy scale is likely to resolve these challenges.

Through the development and implementation of these scales, we noted that climate change and infectious disease literacy, in particular with regards to prevention measures and the dissemination of information, is deficient in LMICs such as Suriname. Our survey data indicated that at least half of the respondents were aware that climate change played a role in infectious disease transmission and were able to associate temperature and precipitation with increased VBDs but did not know exactly how they were spread. We also noted that many participants reported rarely or never participating in basic safety measures to reduce VBD risk, although they agreed that climate change was directly linked to VBD transmission. It is abundantly clear that climate change and VBD literacy should be a more pertinent issue for vulnerable populations as nearly all women in our study population agreed that greater climate change and infectious disease education was needed in their communities (99.7% and 98%, respectively).

Very few climate change and VBD literacy studies have focused their efforts in the Caribbean. We noted from our prior review that the only climate change literacy survey we found that was conducted in the Caribbean came from Jamaica and was very specific to that country [10]. For this reason, we focused our initial efforts on Suriname, with plans to expand our efforts into other Caribbean and South American countries as well as parts of the southern United States. These areas are some of the most affected by climate change and VBDs [43]. Evident changes in the response to climate change in areas of the Caribbean include more intense weather and precipitation patterns, more droughts, more frequent temperature extremes, and increases in sea levels [43]. These changes, if left unmitigated, will very likely continue to impact VBDs through longer transmission seasons as well as increased geographical ranges [44]. In South America specifically, climate suitability for infectious diseases is at an all-time high, with a 35.3% increase in dengue transmission between 2012 and 2021 compared with the 1950–1961 baseline [45].

The creation of a comprehensive climate literacy scale such as ours that expressly addresses important interactions between climate change effects and vector-borne diseases is important for future global health education; there is currently no standardized climate change literacy scale available to researchers to adequately assess climate change and VBD literacy as related topics. At present, many prior studies focus on either climate change or VBD literacy [9] but do not necessarily evaluate increased VBD transmission or vector ranges as consequences of climate change, although these issues are highly correlated. Additionally, previous research has trended towards evaluations of climate change literacy among more highly educated groups such as medical students or healthcare workers, but not always among more vulnerable or high-risk populations such as those living in LMICs [9]. The adaptation of these surveys to the general population will allow us to assess the level of climate change literacy that currently exists within the rest of the population. These scales have the potential to advance climate change and VBD literacy through improving community engagement projects as well as building capacity for climate change and VBD preventive practices in highly vulnerable regions. The scales developed here demonstrate an important step in the advancement of global climate change and VBD literacy not only in the Caribbean, but also in other parts of the world that are also highly affected by climate change and related VBD effects.

Future research includes the cultural adaptation of the scale for implementation in other Caribbean countries such as the Bahamas and Trinidad and Tobago. We also plan to deploy the scale among a larger general population of both men and women in Suriname in order to generate responses from a more representative sample of the population. Lastly, with an entirely female sample, gender differences were not possible to analyze at this point. Sociodemographic outcomes, including gender differences, would be important to assess with the next iteration of scale implementation.

## 5. Conclusions

In all, our validated climate change and VBD scales provide an excellent starting point with regards to evaluating general knowledge, attitudes, and behaviors towards climate change and associated effects, in particular vector-borne disease transmission. This survey instrument is a useful pilot tool that can be utilized to shape future survey instruments and determine specific knowledge gaps in current climate change education in LMICs, especially among more vulnerable populations. Our scales, as described here, can effectively be used to evaluate the need for public health interventions, educational programs, or community engagement efforts, which are essential to increase climate change and VBD literacy in these regions.

## Figures and Tables

**Table 1:** Participant demographics for the exploratory and confirmatory samples.

Characteristic	Exploratory Sample Total, *n* (%)	Confirmatory Sample Total, *n* (%)
**Total**	180 (100)	121 (100)
Average Age (Years)	34.3 ± 6.2	34.5 ± 5.9
District Residence		
**Paramaribo**	129 (72)	82 (68)
**Nickerie**	51 (28)	39 (32)
Race/Ethnicity		
**Chinese**	0 (0)	0 (0)
**Creole**	18 (10)	25 (21)
**Hindustani**	50 (28)	33 (27)
**Javanese**	5 (3)	2 (2)
**Indigenous/Amerindian**	18 (10)	9 (7)
**Caucasian**	0 (0)	0 (0)
**Tribal**	24 (13)	9 (7)
**Two or more reported**	65 (36)	43 (36)
Highest Level of Education		
**None**	9 (5)	4 (3)
**Primary School**	33 (18)	22 (18)
**Lower Secondary School**	39 (22)	36 (30)
**Technical Vocational Training**	2 (1)	0 (0)
**Secondary School**	65 (36)	36 (30)
**Bachelor’s/Master’s**	32 (18)	23 (19)

**Table 2:** Factor loadings and retained items from the climate change EFA.

Item	General	Heat	Cooling	Oceans
**General**				
1. Climate change is mainly caused by humans.	0.867	−0.162	0.174	
2. I believe that climate change will negatively affect future generations.	0.414	0.111		
3. I believe that climate change can be avoided or reduced.	0.560		−0.137	
**Heat**				
4. I believe that heatwaves will happen more often in my area in the future.		0.567		
5. Heatwaves will cause more heatstroke and other heat-related illnesses.	−0.176	0.626		
6. Droughts will become worse because of climate change.		0.541		
7. I believe droughts will become an issue in my area.	0.138	0.411		0.194
**Cooling**				
8. I stay indoors during heatwaves.	0.110	−0.183	0.570	
9. I keep my house as cool as possible during heatwaves.	0.103		0.810	
**Oceans**				
10. The warming of the oceans will negatively affect fish and other sea animals.				0.903
11. The warming of the oceans will make it harder for fishing businesses to make money.	−0.146			0.895

**Table 3:** Eigenvalues for the four constructs retained from the EFA for the Climate Change Scale.

General	Heat	Cooling	Oceans
2.46	2.01	1.77	4.30

**Table 4:** Factor loadings and retained items from the EFA for the Infectious Disease Scale.

Item	Transmission	Temperature	Viruses	Water Containers
**Transmission**				
1. I think there is a direct link between climate change and infectious disease transmission.	0.403		−0.186	
2. Areas that do not currently have disease-carrying mosquitoes may have these mosquitoes in the future.	0.408		−0.163	−0.159
3. Changes in rainfall patterns will increase the spread of infectious diseases.	0.605			
**Temperature**				
4. Higher temperatures will increase the spread of infectious diseases.		0.855		
5. Climate change will increase the chances of catching an infectious disease in warmer countries.		0.699		
**Viruses**				
6. I have noticed that dengue/Zika/chikungunya outbreaks are happening more often in my area.			0.646	
7. I believe that my chances of getting dengue/Zika/chikungunya are greater than other people in my area.			0.648	
**Water Containers**				
8. I remove any water from open containers around my home (e.g., flowerpots, vases, tires, other containers).				0.996
9. I remove things that can hold water outside my house (flowerpots, vases, tires, other containers).	0.122			0.909

**Table 5:** Eigenvalues for the four constructs retained from the EFA for the Infectious Disease Scale.

Transmission	Temperature	Viruses	Water-Holding Containers
1.41	1.93	1.57	2.94

## Data Availability

The data presented in this study are available on request from the corresponding author. The R code used to conduct all analyses can be found at https://github.com/matlackm/climate-literacy/tree/main.

## Supplementary Materials

The following supporting information can be downloaded at: https://www.mdpi.com/article/10.3390/ijerph20247178/s1, Table S1: Classification of literacy items for our general climate change scale by domain (knowledge, attitude, or behavior), Table S2: Classification of literacy items for our infectious disease scale by domain (knowledge, attitude, or behavior), Table S3: Items retained for climate change and infectious disease literacy scale based on 80% or better percent agreement with an essential rating (initial review), or at least 67% percent agreement with an essential or useful but not essential rating (second review) across a panel of six expert reviewers, File S1: Climate and Health Questionnaire for Women of the Caribbean Consortium for Research in Environmental and Occupational Health-MeKiTamara Cohort.

## Appendix A

### Appendix A.1

Scree plots from the exploratory factor analyses for both the Climate Change and Infectious Disease Scales.

### Appendix A.2

Additional tables from the exploratory and confirmatory factor analyses for both the Climate Change and Infectious Disease Scales.

**Table A1:** Inter-factor correlations for the four factors of the Climate Change Scale.

	General	Heat	Cooling	Oceans
**General**	1.00	−0.43	0.35	−0.41
**Heat**	−0.43	1.00	−0.20	0.29
**Cooling**	0.35	−0.20	1.00	−0.07
**Oceans**	−0.41	0.29	−0.07	1.00

**Table A2:** Inter-factor correlations for the four factors of the Infectious Disease Scale.

	Transmission	Temperature	Viruses	Water Containers
**Transmission**	1.00	0.38	0.38	0.17
**Temperature**	0.38	1.00	0.34	0.08
**Viruses**	0.38	0.34	1.00	0.23
**Water Containers**	0.17	0.08	0.23	1.00

**Table A3:** Measures of additional goodness of fit for both the Climate Change and Infectious Disease CFA models.

	Climate Change CFA	Infectious Disease CFA
**Comparative Fit Index (CFI)**	0.99	1.00
**Tucker–Lewis Index (TLI)**	0.99	1.01
**Normed Fit Index (NFI)**	0.85	0.95
**Non-Normed Fit Index (NNFI)**	0.99	1.01
**Relative Fit Index (RFI)**	0.78	0.91
**Incremental Fit Index (IFI)**	0.99	1.01
