# Supplementary material for: Development of a Pilot Literacy Scale to Assess Knowledge, Attitudes, and Behaviors towards Climate Change and Infectious Disease Dynamics in Suriname
Source: Int J Environ Res Public Health. 2023 Dec 14;20(24):7178. doi: 10.3390/ijerph20247178 (PMC10742764; doi:10.3390/ijerph20247178)
Supplement: Supplementary file 1 [file ijerph-20-07178-s001.zip › Supplemental File S1 (questionnaire).pdf]

Climate and Health Questionnaire for Women of the Caribbean Consortium for Research in Environmental and Occupational Health - MeKiTamara Cohort

---

This questionnaire has two sections:

Section A: Demographics and General Information

Section B: Climate Change and Infectious Disease Assessments

Section A: Demographics and General Information

Basic participant information

1. Participant ID

____

2. Recruiter Initials

____

3. Date of Interview (dd/mm/yyyy)

_____/_____/__________

4. Date of Birth (dd/mm/yyyy)

_____/_____/__________

5. Marital Status

   ☐ Married

   ☐ Concubine

   ☐ Separated/Divorced

   ☐ Widow

   ☐ Single

6. Ethnicity (check all that apply)

☐ Chinese

☐ Creole

☐ Hindustani

☐ Indigenous/Amerindian

☐ Javanese

☐ Caucasian

☐ Tribal

☐ Other (please specify) ______________________________

7. In which district do you live?

☐ Paramaribo

☐ Nickerie

☐ Wanica

☐ Saramacca

☐ Commewijne

☐ Para

☐ Coronie

☐ Other (please specify) ________________________

7a. In which village/resort do you live now? (Paramaribo)

- ☐ Beekhuizen
- ☐ Blauwgrond
- ☐ Centrum
- ☐ Flora
- ☐ Latour
- ☐ Livorno
- ☐ Munder
- ☐ Pontbuiten
- ☐ Rainville
- ☐ Tammenga
- ☐ Weg naar Zee
- ☐ Welgelegen

7b. In which village/resort do you live now? (Nickerie)

- ☐ Groot Henar
- ☐ Nieuw-Nickerie
- ☐ Oostelijke Polders
- ☐ Wageningen
- ☐ Westilijke Polders

7c. In which village/resort do you live now? (Wanica)

- ☐ De Nieuwe Grond
- ☐ Domburg
- ☐ Houttuin
- ☐ Koewarasan
- ☐ Kwatta
- ☐ Lelydorp
- ☐ Saramacca Polder

7d. In which village/resort do you live now? (Saramacca)

☐ Calcutta

☐ Groningen

☐ Jarikaba

☐ Kampang Baroe

☐ Tijgerkreek

☐ Wayamboweg

7e. In which village/resort do you live now? (Commewijne)

☐ Alkmaar

☐ Bakie

☐ Margaretha

☐ Meerzorg

☐ Nieuw-Amsterdam

☐ Tamanredjo

7f. In which village/resort do you live now? (Para)

☐ Bigi Poika

☐ Carolina

☐ Noord

☐ Oost

☐ Zuid

7g. In which village/resort do you live now? (Coronie)

☐ Johanna Maria

☐ Totness

☐ Welgelegen

8. Have you moved in the last twelve months?
   - ☐ Yes
   - ☐ No

   8a. If yes, in which village/resort did you live in before?

   ____

9. What is your highest level of education completed?
   - ☐ None
   - ☐ Primary school
   - ☐ Lower secondary school
   - ☐ Technical vocational training
   - ☐ Secondary school
   - ☐ Higher education (Bachelors/Masters)
   - ☐ Other (please specify) __________________________

10. What is your employment status? (please check all that apply)
    - ☐ Employed for wages
    - ☐ Self-employed
    - ☐ Unemployed – looking for work
    - ☐ Unemployed – not looking for work
    - ☐ Homemaker/stay at home parent
    - ☐ Student
    - ☐ Retired
    - ☐ Unable to work due to disability
    - ☐ Other

    10a. If working, do you:
      - ☐ Work in-person only
      - ☐ Work remotely only
      - ☐ Work both in-person and remotely

Housing information

11. How many people currently live in your home (including you)?

____

12. How long have you lived in your current home? (in months)

____

13. What type of housing unit do you live in:

    ☐ Apartment

    ☐ House

    ☐ Other (please specify) ______________________

Outdoor areas/surroundings

14. Do you live close to a body of water (pond, marsh, river, ditch)?

    ☐ Yes

    ☐ No

15. Does the area where you live flood often?

    ☐ Yes

    ☐ No

    15a. If yes, how often does it flood?

        ☐ Never

        ☐ Rarely

        ☐ Sometimes

        ☐ Frequently

        ☐ Always

16. Do you have standing water in your yard after it floods?

☐ Yes

☐ No

16a. If yes, how long does standing water stay in your yard?

☐ less than 1 day

☐ 1-2 days

☐ 3-4 days

☐ 5-6 days

☐ more than 1 week

17. Do you collect rainwater?

☐ Yes

☐ No

18. How often do you collect rainwater during dry seasons?

☐ every day

☐ once a week

☐ twice per month

☐ every month

☐ when it rains

19. How often do you collect rainwater during rainy seasons?

☐ every day

☐ once a week

☐ twice per month

☐ every month

☐ when it rains

20. Is the collected rainwater covered?

☐ Yes

☐ No

21. How long do you let rainwater stand before using?
   - ☐ less than a day
   - ☐ 1-2 days
   - ☐ 3-4 days
   - ☐ 5-6 days
   - ☐ 1-2 weeks
   - ☐ more than 2 weeks

22. Do you use rainwater for cooking?
   - ☐ Yes
   - ☐ No
23. Do you use rainwater for drinking?
   - ☐ Yes
   - ☐ No
24. Do you use rainwater for bathing?
   - ☐ Yes
   - ☐ No
25. Do you use rainwater for housework?
   - ☐ Yes
   - ☐ No

   25a. If yes, what type of housework?
      - ☐ Washing clothes
      - ☐ Washing dishes
      - ☐ Watering plants inside the home
      - ☐ Watering the yard
      - ☐ Cleaning floors
      - ☐ Cleaning the house
      - ☐ Other

26. About how many open standing water sources are in or around your home (e.g. flower pots, houseplants, buckets, vases, car tires, or other open containers that may hold water)?

- ☐ 0 – 4
- ☐ 5 – 9
- ☐ 10 – 14
- ☐ 15 – 20
- ☐ more than 20

27. How often do you leave your doors or windows open during the day?

- ☐ Never
- ☐ Rarely
- ☐ Sometimes
- ☐ Frequently
- ☐ Always

Section B. Climate Change and Infectious Disease Assessments

Please select one answer for each question.

1. Climate change is mainly caused by humans.

Strongly Agree Agree Don't Know Disagree Strongly Disagree

2. Science has shown that climate change is real.

Strongly Agree Agree Don't Know Disagree Strongly Disagree

3. I believe that climate change has an effect on human health.

Strongly Agree Agree Don't Know Disagree Strongly Disagree

4. I believe that climate change has an effect on the environment.

Strongly Agree Agree Don't Know Disagree Strongly Disagree

5. I believe that climate change will negatively affect future generations.

Strongly Agree Agree Don't Know Disagree Strongly Disagree

6. I believe that climate change can be avoided or reduced.

Strongly Agree Agree Don't Know Disagree Strongly Disagree

7. I think there is a direct link between climate change and infectious disease transmission.

Strongly Agree Agree Don't Know Disagree Strongly Disagree

8. Areas that do not currently have disease-carrying mosquitoes may have these mosquitoes in the future.

Strongly Agree Agree Don't Know Disagree Strongly Disagree

9. I am concerned that climate change is increasing the number of mosquitoes that carry infectious diseases in my area.

Strongly Agree Agree Don't Know Disagree Strongly Disagree

10. Climate change will cause the world to become hotter.

Strongly Agree Agree Don't Know Disagree Strongly Disagree

11. Higher temperatures will increase the spread of infectious diseases.

Strongly Agree Agree Don't Know Disagree Strongly Disagree

12. Climate change will increase the chances of catching an infectious disease in warmer countries.

Strongly Agree Agree Don't Know Disagree Strongly Disagree

13. Hotter temperatures across the world will cause more heatwaves.

Strongly Agree Agree Don't Know Disagree Strongly Disagree

14. I believe that heatwaves will happen more often in my area in the future.

Strongly Agree Agree Don't Know Disagree Strongly Disagree

15. Heatwaves will cause more heatstroke and other heat-related illnesses.

Strongly Agree Agree Don't Know Disagree Strongly Disagree

16. I stay indoors during heatwaves.

Never Rarely Sometimes Frequently Always

17. I keep my house as cool as possible during heatwaves.

Never Rarely Sometimes Frequently Always

18. Droughts will become worse because of climate change.

Strongly Agree Agree Don't Know Disagree Strongly Disagree

19. I believe droughts will become an issue in my area.

Strongly Agree Agree Don't Know Disagree Strongly Disagree

20. I store water to use before a drought or other extreme weather event occurs.

Never Rarely Sometimes Frequently Always

21. I reduce the amount of water I use to water my yard or plants during droughts.

Never Rarely Sometimes Frequently Always

22. Climate change will cause more rainfall around the world.

Strongly Agree Agree Don’t Know Disagree Strongly Disagree

23. Changes in rainfall patterns will increase the spread of infectious diseases.

Strongly Agree Agree Don’t Know Disagree Strongly Disagree

24. Climate change will cause more extreme weather, like hurricanes.

Strongly Agree Agree Don’t Know Disagree Strongly Disagree

25. Hurricanes will become worse because of climate change.

Strongly Agree Agree Don’t Know Disagree Strongly Disagree

26. I believe that my area will experience more extreme weather, like hurricanes, because of climate change.

Strongly Agree Agree Don’t Know Disagree Strongly Disagree

27. I keep emergency supplies on hand in case of a hurricane.

Never Rarely Sometimes Frequently Always

28. Climate change will cause sea levels to rise.

Strongly Agree Agree Don’t Know Disagree Strongly Disagree

29. I am worried about sea level rise negatively affecting my community in the future.

Strongly Agree Agree Don’t Know Disagree Strongly Disagree

30. The warming of the oceans will negatively affect fish and other sea animals.

Strongly Agree Agree Don’t Know Disagree Strongly Disagree

31. The warming of the oceans will make it harder for fishing businesses to make money.

Strongly Agree Agree Don’t Know Disagree Strongly Disagree

32. Climate change will negatively impact our ability to grow food.

Strongly Agree Agree Don’t Know Disagree Strongly Disagree

33. Climate change will make it harder to get access to food.

Strongly Agree Agree Don’t Know Disagree Strongly Disagree

34. I would like to reduce the effects of climate change in my area.

Strongly Agree Agree Don’t Know Disagree Strongly Disagree

35. I participate in activities related to reducing climate change effects to help the environment.

Never Rarely Sometimes Frequently Always

36. I believe that there should be more climate change information and education for people in my community.

Strongly Agree Agree Don’t Know Disagree Strongly Disagree

37. I believe that the media should raise awareness about how people can protect themselves from consequences of climate change.

Strongly Agree Agree Don’t Know Disagree Strongly Disagree

38. Mosquitoes prefer temperatures between 24 and 28 degrees Celsius.

Strongly Agree Agree Don’t Know Disagree Strongly Disagree

39. Dengue/Zika/chikungunya are caused by viruses.

Strongly Agree Agree Don’t Know Disagree Strongly Disagree

40. Mosquitoes that can spread dengue/Zika/chikungunya bite more often in the daytime.

Strongly Agree Agree Don't Know Disagree Strongly Disagree

41. There are more dengue/Zika/chikungunya outbreaks during rainy seasons.

Strongly Agree Agree Don't Know Disagree Strongly Disagree

42. I have noticed that dengue/Zika/chikungunya outbreaks are happening more often in my area.

Strongly Agree Agree Don't Know Disagree Strongly Disagree

43. I believe that my chances of getting dengue/Zika/chikungunya are greater than other people in my area.

Strongly Agree Agree Don't Know Disagree Strongly Disagree

44. I feel responsible for preventing dengue/Zika/chikungunya in my area.

Strongly Agree Agree Don't Know Disagree Strongly Disagree

45. I use mosquito nets over my beds while sleeping at home.

Never Rarely Sometimes Frequently Always

46. I use mosquito screens on the doors and windows of my home.

Never Rarely Sometimes Frequently Always

47. I remove any water from open containers around my home (flowerpots, vases, tires, other containers).

Never Rarely Sometimes Frequently Always

48. I remove things that can hold water outside my house (flowerpots, vases, tires, other containers).

Never Rarely Sometimes Frequently Always

49. I make sure my home's septic tank is properly covered.

Never Rarely Sometimes Frequently Always

50. I have reported mosquitoes or mosquito larvae that I see near my house to the appropriate authorities.

Never Rarely Sometimes Frequently Always

51. I think that current control programs offered by the government have been successful in reducing the number of mosquitoes in this area.

Strongly Agree Agree Don't Know Disagree Strongly Disagree

52. I believe that there should be more infectious disease information and education for people in my community.

Strongly Agree Agree Don't Know Disagree Strongly Disagree
