# Supplementary material for: Development of a Pilot Literacy Scale to Assess Knowledge, Attitudes, and Behaviors towards Climate Change and Infectious Disease Dynamics in Suriname
Source: Int J Environ Res Public Health. 2023 Dec 14;20(24):7178. doi: 10.3390/ijerph20247178 (PMC10742764; doi:10.3390/ijerph20247178)
Supplement: Supplementary file 1 [file ijerph-20-07178-s001.zip › Supplemental Tables.pdf]

Table S1. Classification of literacy items for our general climate change scale by domain (knowledge, attitude, or behavior).

KNOWLEDGE	ATTITUDE	BEHAVIOR
Climate change is mainly caused by humans.	I believe that climate change has an effect on human health.	I participate in activities related to reducing climate change effects to help the environment.
Science has shown that climate change is real.	I believe that climate change has an effect on the environment.	I stay indoors during heatwaves.
Climate change will cause the world to become hotter.	I believe that climate change will negatively affect future generations	I try to keep my house as cool as possible during heatwaves.
Hotter temperatures across the world will cause more heatwaves.	I believe that climate change can be avoided or reduced	I have emergency supplies ready in case of a hurricane.
Heatwaves will cause more heatstrokes and other heat-related illnesses.	I would like to reduce the effects of climate change in my area.	I store water to use before a drought or other major weather event.
Droughts will become worse because of climate change.	I believe that heatwaves will happen more often in my area in the future.	I reduce the amount of water I use to water my yard or plants during droughts.
Climate change will cause more rainfall around the world.	I believe that droughts will become an issue in my area.	
Climate change will cause more extreme weather, like hurricanes.	I believe that my area will experience more extreme weather, like hurricanes, because of climate change.	
Hurricanes will become worse because of climate change.	I am worried about sea level rise negatively affecting my community in the future.	
Climate change will cause sea levels to rise.	I believe that there should be more climate change information and education for people in my community.	
The warming of the oceans will negatively affect fish and other sea animals.	I believe that the media should raise awareness about how people can protect themselves from consequences of climate change.	
The warming of the oceans will make it harder for fishing businesses to make money.		
Climate change will negatively impact our ability to grow food.		
Climate change will make it more difficult to get access to food.		

Table S2. Classification of literacy items for our infectious disease scale by domain (knowledge, attitude, or behavior).

KNOWLEDGE	ATTITUDE	BEHAVIOR
Areas that do not currently have disease-carrying mosquitoes may have these mosquitoes in the future.	I think there is a direct link between climate change and infectious disease transmission.	I use mosquito nets over my beds while sleeping at home.
Higher temperatures will increase the spread of infectious diseases.	I am concerned that climate change is increasing the number of mosquitoes that carry infectious diseases in my area.	I use mosquito screens on the doors and windows of my home.
Climate change will increase the chances of catching an infectious disease in warmer countries.	I have noticed that dengue/Zika/chikungunya outbreaks are happening more often in my area.	I remove any water from open containers around my home (flowerpots, vases, tires, other containers).
Changes in rainfall patterns will increase the spread of infectious diseases.	I believe that my chances of getting dengue/Zika/chikungunya are greater than other people in my area.	I remove things that can hold water outside my house (flowerpots, vases, tires, other containers).
Mosquitoes prefer temperatures between 24 and 28 degrees Celsius.	I feel responsible for preventing dengue/Zika/chikungunya in my area.	I make sure my home's septic tank is properly covered.
Dengue/Zika/chikungunya are caused by viruses.	I think that current control programs offered by the government have been successful in reducing the number of mosquitoes in this area.	I have reported mosquitoes or mosquito larvae that I see near my house to the appropriate authorities.
Mosquitoes that can spread dengue/Zika/chikungunya bite more often in the daytime.	I believe that there should be more infectious disease information and education for people in my community.	
There are more dengue/Zika/chikungunya outbreaks during rainy seasons.		

Table S3. *Items retained for climate change and infectious disease literacy scale based on 80% or better percent agreement with an essential rating (initial review), or at least 67% percent agreement with an essential or useful but not essential rating (second review) across a panel of six expert reviewers. This left a total of 52 items for exploratory and confirmatory analyses.*

	**Rating**	**Percent Agreement**
**1. Climate change is mainly caused by humans.**	Essential (E)	100%
**2. Science has shown that climate change is real.**	Essential (E)	83.3%
**3. I believe that climate change has an effect on human health.**	Essential (E)	100%
**4. I believe that climate change has an effect on the environment.**	Essential (E)	83.3%
**5. I believe that climate change will negatively affect future generations.**	Essential (E)	83.3%
**6. I believe that climate change can be prevented or avoided.**	Essential (E)	100%
**7. I think there is a direct link between climate change and infectious disease transmission.**	Essential (E)	83.3%
**8. Areas that do not currently have disease-carrying mosquitoes may have these mosquitoes in the future.**	Essential (E)	83.3%
**9. I am concerned climate change is increasing the number of mosquitoes that carry infectious diseases in my area.**	Essential (E)	83.3%
**10. Climate change will cause the world to become hotter.**	Essential (E)	83.3%
**11. Higher temperatures will increase the spread of infectious diseases.**	Essential (E)	100%
**12. Climate change will increase the chances of catching an infectious disease in warmer countries.**	Essential (E)	83.3%
**13. Hotter temperatures across the world will cause more heatwaves.**	Essential (E)	83.3%
**14. I believe that heatwaves will happen more often in my area.**	Essential (E)	83.3%

**15. Heatwaves will cause more heatstroke and other heat-related illnesses.**	Essential (E)	83.3%
**16. I stay indoors during heatwaves.**	Useful but not essential (U)	67%
**17. I keep my house as cool as possible during heatwaves.**	Useful but not essential (U)	83.3%
**18. Droughts will become worse because of climate change.**	Essential (E)	100%
**19. I believe that droughts will become an issue in my area.**	Essential (E)	83.3%
**20. I store water to use before a drought or other extreme weather event occurs.**	Useful but not essential (U)	67%
**21. I reduce the amount of water I use to water my yard or plants during droughts.**	Useful but not essential (E)	67%
**22. Climate change will cause more rainfall around the world.**	Essential (E)	83.3%
**23. Changes in rainfall patterns will increase the spread of infectious diseases.**	Essential (E)	83.3%
**24. Climate change will cause more extreme weather, like hurricanes.**	Essential (E)	100%
**25. Hurricanes will become worse because of climate change.**	Essential (E)	100%
**26. I believe that my area will experience more extreme weather, like hurricanes, because of climate change.**	Essential (E)	83.3%
**27. I have emergency supplies ready in case of a hurricane.**	Essential (E)	83.3%
**28. Climate change will cause sea levels to rise.**	Essential (E)	100%
**29. I am worried about sea level rise negatively affecting my community.**	Essential (E)	83.3%
**30. The warming of the oceans will negatively affect fish and other sea animals.**	Essential (E)	83.3%
**31. The warming of the oceans will make it harder for fishing businesses to make money.**	Useful but not essential (U)	67%
**32. Climate change will negatively impact our ability to grow food.**	Essential (E)	83.3%

**33. Climate change will make it harder to get access to food.**	Essential (E)	83.3%
**34. I would like to reduce the effects of climate change in my area.**	Essential (E)	83.3%
**35. I participate in activities related to reducing climate change effects to help the environment.**	Essential (E)	83.3%
**36. I believe that there should be more climate change information and education for people in my community.**	Essential (E)	83.3%
**37. I believe that the media should raise awareness about how people can protect themselves from consequences of climate change.**	Essential (E)	83.3%
**38. Mosquitoes prefer temperatures between 24 and 28 degrees Celsius.**	Useful but not essential (U)	83.3%
**39. Dengue/Zika/chikungunya are caused by viruses.**	Useful but not essential (U)	67%
**40. Mosquitoes that can spread dengue/Zika/chikungunya bite more often in the daytime.**	Useful but not essential (U)	83.3%
**41. There are more dengue/Zika/chikungunya outbreaks during rainy seasons.**	Essential (E)	83.3%
**42. I have noticed that dengue/Zika/chikungunya outbreaks are happening more often in my area.**	Useful but not essential (U)	67%
**43. I believe that my chances of getting dengue/Zika/chikungunya are greater than other people in my area.**	Useful but not essential (U)	67%
**44. I feel responsible for preventing dengue/Zika/chikungunya in my area.**	Useful but not essential (U)	67%
**45. I use mosquito nets over my beds while sleeping at home.**	Essential (E)	83.3%
**46. I have mosquito screens on the doors and windows of my home.**	Essential (E)	83.3%

**47. I remove any water from open containers around my home (flowerpots, vases, tires, other containers).**	Essential (E)	83.3%
**48. I remove things that can hold water outside my house (flowerpots, vases, tires, other containers).**	Essential (E)	83.3%
**49. I make sure my home's septic tank is properly covered.**	Essential (E)	83.3%
**50. I have reported mosquitoes or mosquito larvae that I see near my house to the appropriate authorities.**	Essential (E)	83.3%
**51. I think that current control programs offered by the government have been successful in reducing the number of mosquitoes in this area.**	Useful but not essential (U)	67%
**52. I believe that there should be more infectious disease information and education for people in my community.**	Essential	83.3%
